# Analysis of Correlates and Trends in Caregivers’ Mental Health Pre- and COVID-Onward From the HINTS, 2018-2022

**DOI:** 10.1093/geroni/igaf122.2154

**Published:** 2025-12-31

**Authors:** Jingxin Yao, Xiayu Chen, Sasha Zhou, Minakshi Raj

**Affiliations:** University of Illinois Urbana-Champaign, Urbana, Illinois, United States; University of Central Florida, Urbana, Illinois, United States; Wayne State University, Detroit, Michigan, United States; University of Illinois Urbana-Champaign, Urbana, Illinois, United States

## Abstract

**Objectives:**

To compare correlates of mental distress among caregivers versus non-caregivers, and evaluate trends in caregivers’ mental distress pre- versus COVID-onward.

**Methods:**

This cross-sectional study used nationally representative data from multiple cycles of the Health Information National Trends Survey (2018 to 2022; n = 15,318). We characterized sociodemographic, health, and caregiving characteristics and estimated logistic regression models evaluating correlates of mental distress among the full sample and pre- and COVID-onward.

**Results:**

Caregivers reported a higher mean PHQ-4 score than non-caregivers overall (|t|=2.88, p<.01). Caregiving status was significantly associated with mental distress pre-COVID (OR = 1.47; 95%CI=1.13-1.92; p<.01) and COVID-onward (OR = 1.51; 95%CI=1.01-2.26; p<.05). Being in fair or poor health status was associated with a higher likelihood of mental distress pre-COVID (OR = 2.58, 95%CI=1.31-5.09, p < 0.01) while having a college education or higher was associated with a lower likelihood of mental distress COVID-onward (OR = 0.46, 95%CI=0.21-0.99, p< .05).

**Conclusions:**

Caregiving status continues to be a significant risk factor for mental distress. Factors significantly associated with mental distress among caregivers change over time. Findings suggest the COVID-19 pandemic may continue to present risks to caregivers’ mental wellbeing, requiring development and implementation of policies and programs to address caregiver mental health.

